# Mechanism of Heat-Induced Fusion of Silver Nanowires

**DOI:** 10.1038/s41598-020-66304-2

**Published:** 2020-06-09

**Authors:** Chang-Lae Kim, Joon-Young Lee, Dong-Gap Shin, Jong-Souk Yeo, Dae-Eun Kim

**Affiliations:** 1 Department of Mechanical Engineering, Chosun University, Gwangju, 61452 Republic of Korea; 2 School of Integrated Technology, Yonsei University, Incheon, 21983 Republic of Korea; 3 School of Mechanical Engineering, Yonsei University, Seoul, 03722 Republic of Korea

**Keywords:** Materials science, Nanoscience and technology

## Abstract

Physical changes in arranged silver nanowires were monitored during progressive heating inside a transmission electron microscope. Using the *in-situ* experimental method, overall variation of silver nanowires and movement of the silver atoms could be assessed. The physical morphology of silver nanowires was rapidly transformed above 350 °C as they fused with each other, which led to extrusion of the silver atoms. Around 550 °C, silver nanowires were almost fused into one, filling a relatively large void between silver nanowires. However, above 575 °C, the united silver nanowire was completely cut off, starting from the region that was suspected to have defects. For the first time, the fusion of arranged silver nanowires and the configurational changes of silver atoms during heating were visualized, and the migration between silver atoms and the damage mechanism of silver nanowires were assessed. Moreover, the relationship of physical morphology and electrical property of silver nanowires according to the temperature were investigated using the *ex-situ* experimental method. As silver nanowires started to split at 300 °C, the electrical conductivity deteriorated greatly. Beyond 350 °C, the electrical conductivity was completely lost while silver nanowires disintegrated rapidly, and silver nanowires completely disappeared at 450 °C.

## Introduction

Metal nanowires have received much attention because of their unique physical, chemical, electrical, and mechanical characteristics in various fields such as flexible displays, touch panels, sensors, heaters, and energy devices^1–10^. Various studies on metal nanowire growth technology, synthesis mechanism, mechanical/electrical behaviors, and frictional phenomena have been conducted to adopt them to nanoscale devices^1,11–17^. Especially, in order to improve electrical and mechanical stability of the electrode based on metal nanowires, there have been many attempts to connect junctions of metal nanowires through various methods such as chemical joining, laser nano-welding and nano joining at the junction with carbon nanomaterials^12–17^. In application of metal nanowires, sufficient thermal stability of metal nanowires is needed to form electrical connections of nanoscale devices and for use as building blocks. There have been several studies on the phenomenon of damaged metal nanowires in the form of nanodots caused by Rayleigh instability when exposed to high temperature^18–21^. However, the studies regarding the thermal characteristics and behaviors are still insufficient to fully understand the thermal behavior of metal nanowires. In this regard, understanding the damage mechanism of metal nanowires due to heating is important because it is closely related to the conductivity, durability and performance of metal nanowires.

Studies have been conducted to obtain the optimized conditions for sorting metal nanowires that can tolerate high temperature and for deciding which metal nanowires could be used for the electrical interconnection of nanoscale devices. It was shown that the reason for the significant variation in the electrical resistance of metal nanowires over a certain range of temperature was due to configurational instability^22^. Thermal annealing has been frequently used as an after-treatment process to control the physical and electrical characteristics of metal nanowires^22–25^. It was shown that the electrical conductivity of silver nanowires was enhanced as the sintering and organic residues disappeared while heating to 300 °C through the thermal annealing process^22^. Moreover, it was verified that electrical conductivity was greatly improved by fusion at the contact point of the crossed metal nanowires from annealing or Joule heating^23,24^. However, increased electrical resistance was also noticed due to damage to the metal nanowires upon reaching high temperature and having Rayleigh instability^23,25^. In previous studies, metal nanoparticles were subjected to local heat treatment while minimizing damage by the selective laser sintering process^26–28^. Therefore, there should be a deep understanding of thermal stability to assure high durability and reliability of metal nanowires in nanoscale devices produced and operated at high temperature, and to obtain optimal metal nanowires by reducing metal nanowire defects with after-treatment.

In this study, the thermal characteristics of silver nanowires were investigated using a heating stage installed inside a transmission electron microscope (TEM). Silver nanowires were selected because they are frequently utilized in a variety of nanoscale devices due to their superior chemical and electrical characteristics, as well as corrosion resistance. With several strands of silver nanowires in a parallel arrangement, configurational changes due to increased temperatures, especially by the process of fusion and damage, were visualized in real time. In addition, changes in physical and electrical properties of silver nanowires coated with a large area were evaluated by the *ex-situ* method.

## Materials and Methods

### Materials

Silver nanowire solution was synthesized using ethylene glycol (EG), silver nitrate (AgNO_3_), silver chloride (AgCl), and polyvinyl pyrrolidone (PVP) through the polyol method^1,3,11^. First, a certain amount of PVP (1,300,000 g/mol) powder was dissolved in the EG solution (which played the role of a reductant) with stirring and heating at 170 °C. Next, the PVP-EG solution was in sequence mixed with KBr (119 g/mol) and AgCl (143.32 g/mol) that could reduce the Ag seed generation at the same temperature. Then, a small amount of AgNO_3_ solution (169.87 g/mol) was slowly added to the former solution at a rate of 0.02 ml/sec so that silver nanowire started to grow through reaction at 170 °C for 2 h. The pure silver nanowire could be synthesized after the cooling, cleaning, and centrifugation processes. The silver nanowire used in this study had the FCC crystal structure and the preferred orientation was the (111) direction^1^. In addition, silver nanowires with diameter and length of about 30–80 nm and below 20 µm, respectively, were selected.

### Specimen preparation

After drop casting a drop of silver nanowire solution on a thermal E-chip without a supporting membrane (E-AHBN, Protochips, USA), it was cleaned with ethanol for 5 minutes and dried naturally in the atmosphere for 20 min. The silver nanowires formed on the thermal E-chip were subjected to the ion beam cleaning process, making the solution completely dried. Experiments for tracking and monitoring the configurational changes with temperature were conducted using several strands of silver nanowires hanging on a void in parallel arrangement in the thermal E-chip.

A specimen coated with silver nanowires on a glass substrate (25 mm × 70 mm) was prepared in order to analyze the relationship between physical morphology and electrical conductivity of silver nanowires. Furthermore, a specimen with two crossed silver nanowires on the glass was utilized to visualize the morphological variation of silver nanowires with respect to temperature through the scanning method.

### Experiments

Using a spherical aberration correction scanning transmission electron microscopy (Cs-corrected STEM, JEM-ARM 200F, JEOL, USA), equipped with a CEOS CS corrector on the illumination system, *in-situ* TEM analysis of silver nanowires was performed. *In-situ* heating was performed from RT to 750 °C with 5 °C/s temperature ramp using a heating holder system (Aduro 500, Protochips, USA). High resolution TEM (HR-TEM) images of the free standing silver nanowire were achieved at each temperature after 5 min holding time. To obtain the HR-TEM images, an objective lens with aperture of 50 μm was used.

Using a three-dimensional laser scanning confocal microscopy (3D-LSCM, VK-X200, KEYENCE, USA), *ex-situ* 3D-LSCM analysis of silver nanowires was carried out. The silver nanowire coating deposited on the glass substrate (25 mm × 70 mm) was placed on a hot plate and heated at 50 °C intervals from 100 °C to 450 °C under atmospheric conditions. The morphological variation of silver nanowires at each temperature was observed through the 3D-LSCM. During the variation process of morphology of silver nanowires, the electrical properties of silver nanowires were analyzed using a 4-point probe electrical measurement system (Keithley 2001 digital multimeter, Keithley, USA).

Using a high resolution atomic force microscopy (HR-AFM, NX-10, Park Systems, Korea), *in-situ* AFM analysis of silver nanowires was conducted. In order to visualize the shape of two crossed silver nanowires with respect to temperature, *in-situ* heating and *in-situ* scanning were performed in real time using a heating plate and an AFM probe (PPP-NCHR tip, NANOSENSORS, Switzerland) at the same points.

## Results and Discussion

**Figure 1:**
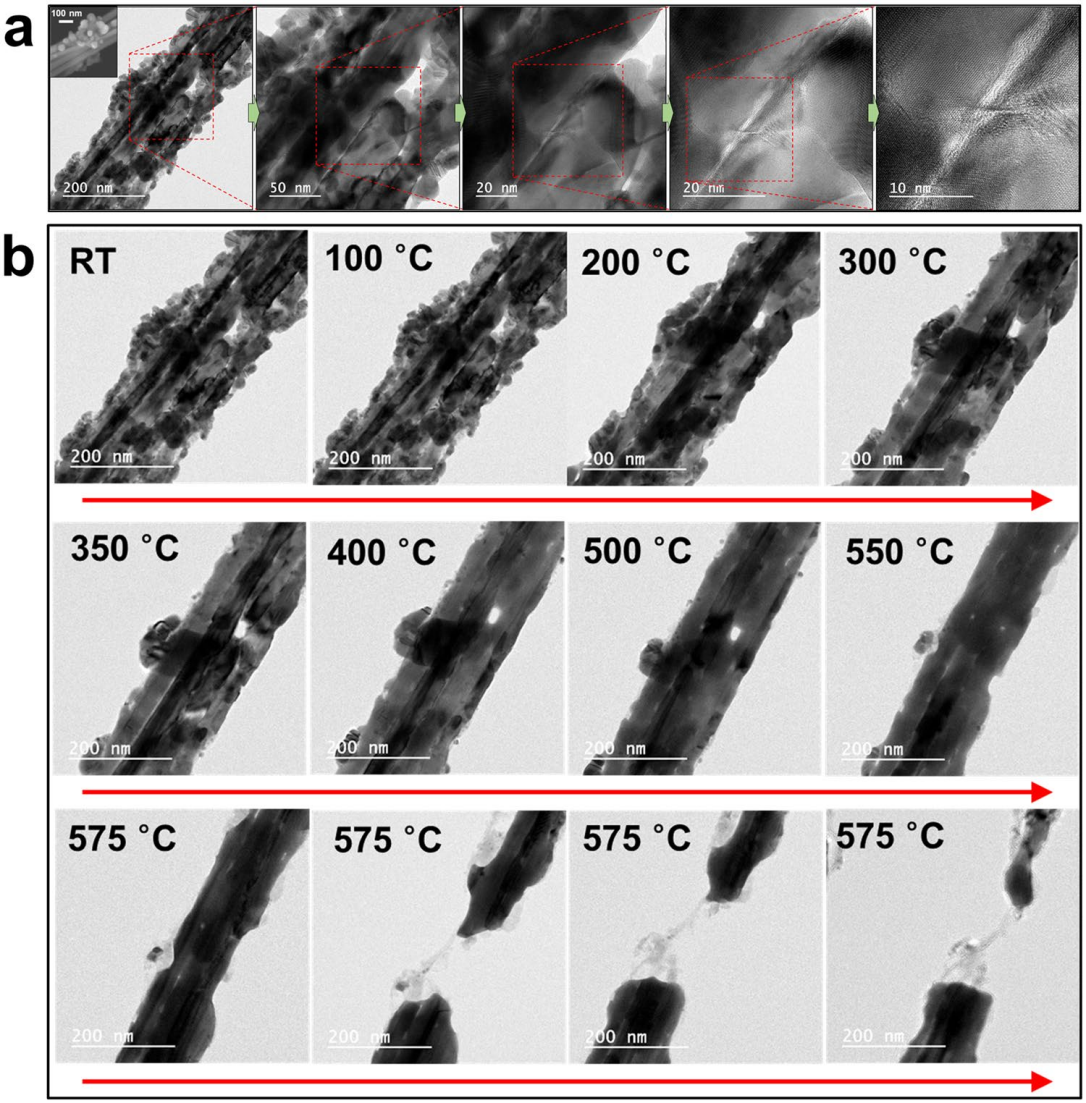
TEM images of parallel strands of silver nanowires. (**a**) Representative TEM images of parallel strands of silver nanowires from low magnification to high. Inset: SEM image of parallel strands of silver nanowires. Silver nanoparticles are attached on silver nanowires. Clear boundaries between nanowires and a large void between the strands of nanowires are observed. TEM image at high magnification shows the arrangement of atoms in the crystal structure. (**b**) *In-situ* monitored TEM images of silver nanowires at different temperatures from RT to 575 °C. No significant change of the morphology of silver nanowires at 100 °C. Nanoparticles on silver nanowires were fused and shrunk between 200 °C and 300 °C. The size of fused particles was significantly reduced from 350 °C to 500 °C. The void at the center was blocked at 550 °C. The fused silver nanowire was completely broken at 575 °C.

Silver nanowires were synthesized with about 30–80 nm diameters and below 20 µm lengths (Fig. 1a). Nanoparticles generated from the synthesis process were attached on the surfaces of silver nanowires. Most silver nanowires were rather straight, but the longer ones were bent in various angles and some of them had neck formations. Some parallel strands of silver nanowires were selected for observation from among silver nanowires hanging on the opening of a heating holder (Fig. 1a). TEM analysis was conducted at room temperature (RT), particularly at the region between the strands of silver nanowires (Fig. 1a). There was apparent evidence of bunched silver nanowires where several clear lines between silver nanowires and the large void in the region between strands of silver nanowires could be observed. The arrangement of atoms in the crystal structure could be found from the TEM images at high magnification (Fig. 1a).

To analyze the effect of temperature on silver nanowires, a thermal E-chip without supporting membrane (E-AHBN, Protochips, USA) was installed inside the TEM^29–32^. The maximum temperature of 1200 °C could be achieved while controlling the rate of temperature increase. Figure 1b shows the *in-situ* TEM images of the variation of the morphology of silver nanowires and nanoparticles as the temperature was increased from RT. When heated to 100 °C, there was no significant change in the shape of silver nanowires relative to RT. This indicated that silver nanowires had sufficient thermal stability at 100 °C, which was lower than the heating temperature of 160–180 °C applied during the synthesis process^11^. The physical modification of nanoparticles initiated at a relatively low temperature. As the temperature increased to 200 °C, nanoparticles that were attached unevenly to the surfaces of silver nanowires started fusing to generate a slightly smooth shape^33–36^. The volume of fused particles shrank around 300 °C, and the particles on the surface eventually disappeared. It was inferred that the nanoparticles were absorbed into silver nanowires. In other words, silver atoms in the nanoparticles with relatively small size diffused in a high temperature environment and moved inside silver nanowires to which they were attached to^37–40^. Nanoparticles fused during the process of increasing the temperature from 350 °C to 500 °C and the size of agglomerated particles shrank rapidly, making it about ten times smaller compared to the original size before heating. On the other hand, it is also speculated that the atoms of the nanoparticles slowly evaporated to the surroundings. This was considered to be due to the surface diffusion and/or evaporation of silver atoms^37–40^. It was found that the resolution of the boundary, which was indicated by the strong contrasts of the shades of silver nanowires in TEM images, was lowered and the void (formed in the region between strands of silver nanowires) became smaller. At 550 °C, the void at the center was completely blocked, uniting silver nanowires into one and blurring the boundaries between them. Moreover, it could be seen that the surface of silver nanowires changed. Damaged parts, which resembled etched surface, were found on both the left and right parts from the region between silver nanowires. Upon raising the temperature to 575 °C, the small damaged part started to undergo sudden figural changes and completely broke in dozens of seconds. Both the processes of fusion of originally independent nanoparticles and nanowires, and damage due to temperature increase could be visualized in real time. It was postulated that silver atoms moved in a certain direction due to the temperature increase because of the disappearance of nanoparticles, blockage of voids, and configurational changes of the volume of silver nanowires. This process was analogous to the fracture phenomenon of a silver nanowire incurred during the tensile test in which high-rate atomic diffusion was suggested as the main mechanism for the rapid alteration in the shape of the silver nanowire^41^.

**Figure 2:**
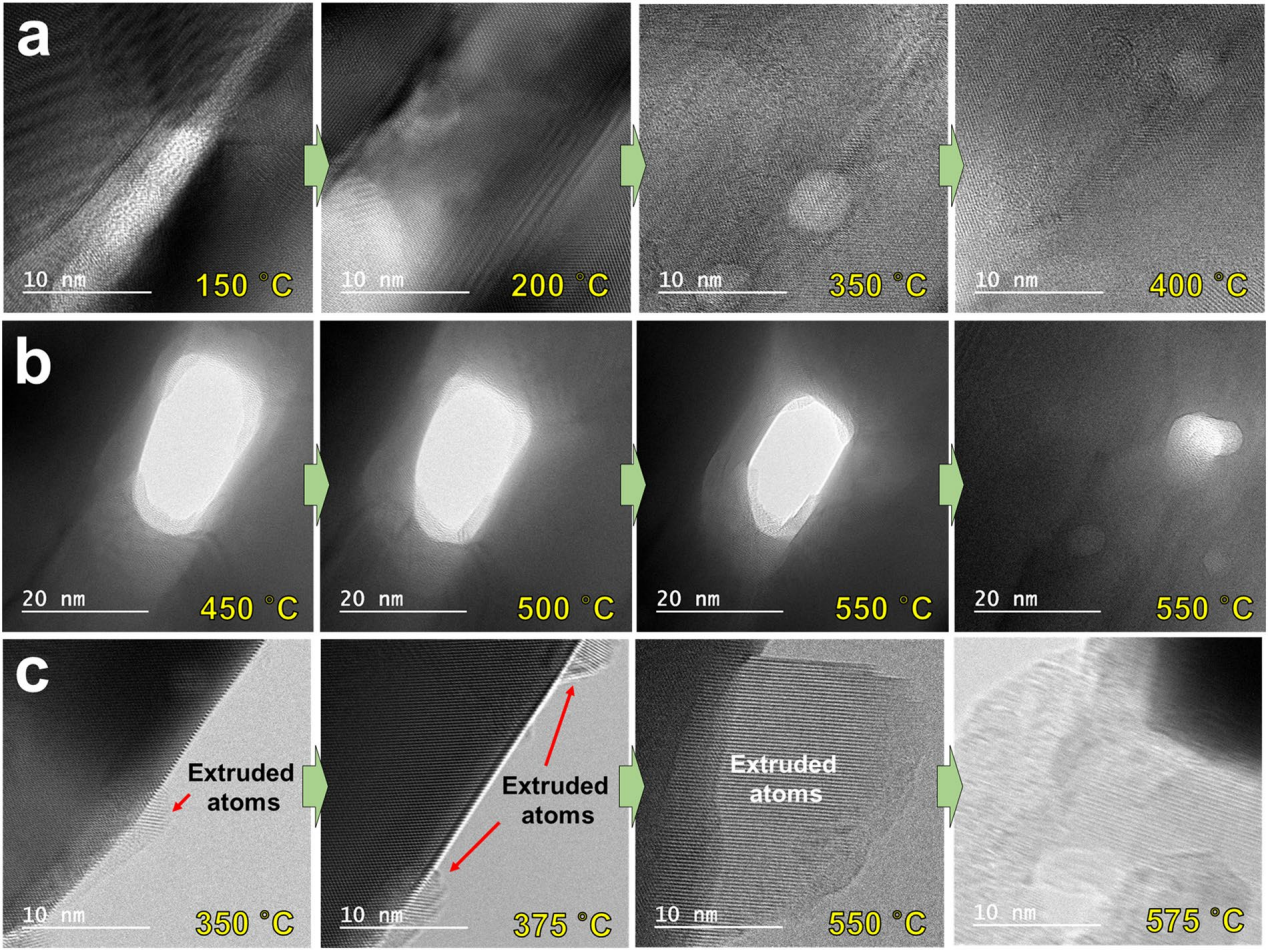
TEM images at high magnification of the configurational changes of silver nanowires at different temperatures. (**a**) Boundaries between nanowires that gradually fade from 150 °C to 400 °C. (**b**) Void between nanowires that gradually filled from 450 °C to 550 °C. (**c**) Silver atoms gradually extruded toward the circumferential direction of silver nanowire from 350 °C to 550 °C.

For detailed analysis of the configurational change of silver nanowires and silver nanoparticles with the increase in temperature, TEM images were obtained at high magnification as shown in Fig. 2. The fusion process in which silver nanowires united into one upon increasing the temperature from 150 °C to 400 °C, could be verified through the TEM images at high magnification (Fig. 2a). In addition, the migration of silver atoms could be demonstrated by monitoring the process that filled the voids between silver nanowires at temperatures between 450 °C and 550 °C (Fig. 2b). Moreover, it was clearly evident that silver atoms moved when the temperature was increased since extruded atoms, which started to appear on the sides of silver nanowires at 350 °C, protruded by about 15–20 nm at 550 °C (Fig. 2c). It was also noticed from the TEM images at high magnification that at 550 °C the protruded atoms were arranged in a uniform direction. It was found that the side parts of the united silver nanowire where atoms protruded were dented toward the inside of the silver nanowire. Furthermore, the end of the arrangement for extruded atoms on the other side (i.e., first protruded atoms) was found to be quite blurry without any specific atomic structure. After breaking the silver nanowires by heating at 575 °C, the TEM images showed that silver atoms vanished due to evaporation^42,43^.

**Figure 3:**
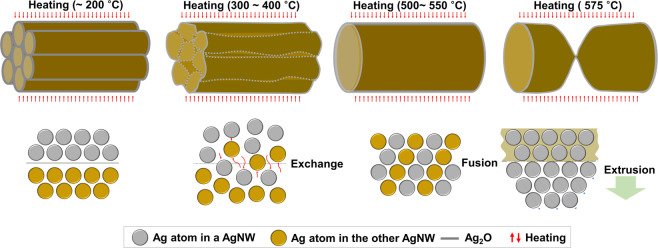
Schematic designs of the atomic migration and fusion mechanisms of silver nanowires. No significant variation until 200 °C. Melting of silver oxide layers at 300–400 °C. Fusion of all strands of nanowires at 500–550 °C. Fracture of fused silver nanowire due to extrusion of silver atoms at 575 °C.

As the temperature increased, the silver nanowires became one by fusion and the thickness of the combined silver nanowire decreased with increasing temperature (Fig. 2). As explained above, this was the result of the migration of atoms by diffusion and evaporation. In particular, diffusion in the solid state is considered to be the main mechanism for the migration of atoms. The level of migration of atoms grew with increasing temperature which initiated from around the central regions of the nanowires where defects existed. Likewise, several findings that explain the mechanism of the migration of atoms in nanowires were previously reported^44–48^. Previous studies showed that the migration of atoms was caused by diffusion of the atoms with high kinetic energy due to rising temperature^44^. In other words, the Kirkendall effect resulting from the different diffusion rates of metallic atoms was the primary mechanism for moving the boundary layer between two metals^45–47^. Another study reported that besides diffusion, the migration of atoms became easier because silver nanowires started melting above a certain temperature^48^. This migration between atoms naturally formed voids, which accelerated atomic movement and eventually contributed to major damage of the silver nanowire structure. Previous studies reported similar results, but they were conducted on encapsulated forms with other elements such as carbon, TiO_2_ shell, and Ag_2_Se on the outside of the silver nanowires. Moreover, only one silver nanowire was used to observe the migration of atoms. However, in this study, the migration and fusion of atoms (caused by temperature increase) between silver nanowires arranged in parallel were visualized for the first time. After the silver nanowires were fused, the entire process from the migration of atoms by diffusion to fracture by defects was monitored in real time and the main mechanism for variation was investigated. The mechanism of atomic migration and fusion is summarized in Fig. 3. First, the overlapping strands of silver nanowires showed good stability without any significant variation below 200 °C. However, at 300–400 °C, oxide layers surrounding each of the silver nanowires started melting (the melting point of Ag_2_O is known to be lower than 300 °C) and the atomic exchange between silver nanowires initiated^49^. Next, at about 500–550 °C all strands of silver nanowires were fused and became one. During this process, atomic extrusion occurred, which caused the atoms to move toward the outside of silver nanowires in a certain direction, resulting in the eventual fracture of silver nanowires by defects. To describe the fracture of silver nanowires by heating, previous studies reported Rayleigh instability of materials such as copper, gold, and tin^18–21^. The lengths of silver nanowires were much longer than the diameters in the circumferential direction and the atoms were unstable in terms of energy, in spite of their regular arrangement. Alteration of silver nanowires initiated at a lower temperature than the bulk materials, and the connections between the silver nanowires started breaking. This came about from the regions that had high migration of atoms by diffusion at an increased temperature, because of the capillary action and surface tension from the melted atoms.

The key mechanism of migration of atoms between silver nanowires was diffusion, causing a direct exchange of atoms^37–40,44–47^. In other words, atoms were switched with other atoms in nearby lattice sites with rising temperature. This suggested that because each atom moved another one in a different direction through the interface, atomic fluxes from the interface between several strands of silver nanowires could be the same. Further, diffusion caused by lattice vacancies could be considered^45,46^. An atom moved to an empty lattice site through the exchange of the space between an atom and a vacancy. In case there was large diffusion within one silver nanowire, the atoms flowed in one direction and the vacancies went the other way. Even though the atoms were extruded toward the outside of silver nanowires and other atoms filled the space originally occupied, the diameter of silver nanowire as a whole remained thin. Moreover, when a huge atomic flux is caused by dislocations and defects of silver atoms generated in the fusion process of silver nanowires and nanoparticles, silver nanowires experienced high level of stress resulting in overall fracture. This fracture at a high temperature was similar to the shape of fractured materials in a mechanical tensile test. That is, the shape of silver nanowires, created by the defects on the surface of the silver nanowires due to extruded atoms, was similar to the shape of a Gaussian specimen (dog-bone specimen) used for the tensile test. The entire silver nanowire was more likely to experience a tensile stress, not by mechanical tension but due to a variety of phenomena such as interaction between atoms inside the silver nanowires, diffusion, extrusion, evaporation, and melting from increased temperature.

**Figure 4:**
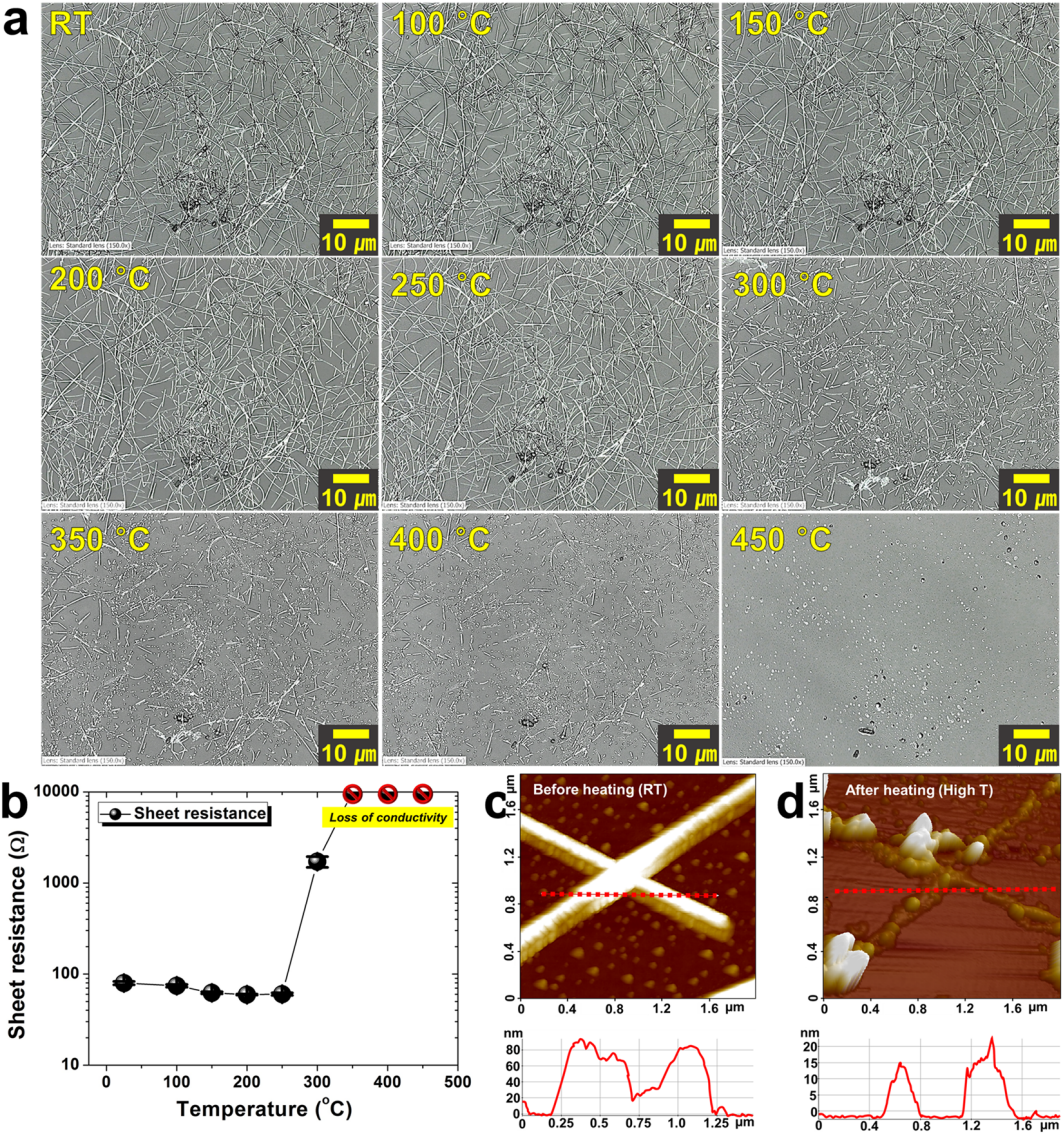
Variation in physical and electrical properties of silver nanowires scattered over a large area depending on temperature. (**a**) *Ex-situ* monitored laser scanning confocal microscope images of silver nanowires at different temperatures from RT to 450 °C. No significant change of the morphology of silver nanowires up to 250 °C. Silver nanowires were split between 250 °C and 300 °C. The length of cleaved silver nanowires was significantly shortened after 350 °C. Silver nanowires completely disappeared at 450 °C. (**b**) Variation of sheet resistance of silver nanowires at different temperatures. The sheet resistance of silver nanowires decreased slightly to 250 °C and then increased significantly at 300 °C. At temperatures above 350 °C, the electrical conductivity of silver nanowires was completely lost. (**c,d**) Scanning images (top) and 2d profile graphs (bottom) of two crossed silver nanowires (**c**) before heating and (**d**) after heating.

The relationship of physical morphology and electrical property of silver nanowires with respect to temperature variation were analyzed as shown in Fig. 4. A specimen coated with silver nanowires on a glass substrate (25 mm × 70 mm) was prepared. The specimen was placed on a hot plate and heated at 50 °C intervals from 100 °C to 450 °C under atmospheric conditions. The morphological variation of silver nanowires at each temperature was observed through three-dimensional laser scanning confocal microscope (3D-LSCM). From RT to 250 °C, it was confirmed that the silver nanowires retained their original shape without any significant change. At 300 °C, silver nanowires started to split. The length of cleaved silver nanowires began to shrink at 350 °C and was significantly shortened at 400 °C. Eventually, the silver nanowires completely disappeared after heating at 450 °C. Unlike the results of *in-situ* TEM experiments in a vacuum environment, the silver nanowires were broken at relatively low temperatures in *ex-situ* experiments conducted in the atmosphere. It was postulated that as the temperature increased in ordinary atmospheric environment, the breakdown progress of silver nanowires was accelerated due to oxidation. Since the arrangement (intersection vs. parallel) of silver nanowires was also different, it was thought that the temperature range that caused morphological variation was different. In addition, if the material of the nanowire is different, the temperature range will also be different. In particular, depending on the heating rate and heating path, the temperature range that causes morphological variation of silver nanowires will vary. In order to clearly understand the mechanism for changing the morphologies and electrical properties of silver nanowires according to temperature, it is necessary to further analyze changes in the characteristics of silver nanowires according to various heating rates and heating paths.

The electrical properties of silver nanowires coated with large area were also evaluated by observing changes in shape with respect to temperature. Figure 4b shows the change in sheet resistance of silver nanowire coating with increasing temperature. The sheet resistance (under 100 Ω) decreased slightly from RT to 250 °C. It was confirmed earlier that there was no significant change in the physical morphology of silver nanowires during this temperature variation stage. The subtle reduction in sheet resistance was expected to be due to the fact that the impurities between silver nanowires disappeared and the silver nanowires were fused to each other to slightly improve the electrical conductivity. However, at 300 °C, as silver nanowires started to split, the sheet resistance (about 1.7 kΩ) was greatly increased, resulting in a significant loss in conductivity. Since silver nanowires were severely damaged from 350 °C, the sheet resistance increased significantly and the electrical conductivity was completely lost. From these results, the degree of efficiency degradation of electrodes and the limitation on thermal durability of silver nanowires in high temperature environment could be assessed.

Figure 4c,d shows the scanning images and 2d profile graphs of two crossed silver nanowires measured with a high resolution atomic force microscope (HR-AFM) at RT before heating (Fig. 4c) and at high temperature after heating (Fig. 4d). *In-situ* AFM measurement was used to scan the same points on the same specimen while increasing the temperature, so that the morphological change of silver nanowires with respect to temperature could be assessed. From the scanning images, it was confirmed that silver nanowires were damaged and lost after being heated. 2d profile graphs show that the thickness of silver nanowires was greatly reduced after heating.

In conclusion, the overall physical changes of silver nanowires in a parallel arrangement and the migration of atoms with respect to temperature were visualized in real time using an *in-situ* TEM experimental method. The physical morphology of nanowires changed rapidly with increasing temperature due to diffusion and extrusion of the atoms and fused with each other. Around 550 °C, silver nanowires were united by fusing but at a critical temperature of 575 °C, the united silver nanowire was damaged. Therefore, in this study the fusion of silver nanowires in a parallel arrangement and the configurational changes of atoms during the heating process were visualized and monitored in real time, demonstrating the main mechanism for the process such as atomic migration and fracture. In addition, the variation of physical morphology and electrical property of silver nanowires depending on temperature were monitored by an *ex-situ* 3D-LSCM experimental method. The sheet resistance of silver nanowires, which started to split at 300 °C, increased greatly. At 350 °C, the electrical conductivity of silver nanowires was completely lost, and the shape of the nanowire completely disappeared at 450 °C. Furthermore, the variation of physical morphology of two crossed silver nanowires at RT and at high temperature was visualized in real time using an *in-situ* AFM experimental method. The silver nanowires became thinner as the nanowires were damaged by heat. The results obtained from analyzing the changes of morphology and sheet resistance of silver nanowires with temperature increase are expected to be helpful in utilizing silver nanowires as transparent electrodes.
